# Skeletal muscle reprogramming by breast cancer regardless of treatment history or tumor molecular subtype

**DOI:** 10.1038/s41523-020-0162-2

**Published:** 2020-06-04

**Authors:** Hannah E. Wilson, David A. Stanton, Cortney Montgomery, Aniello M. Infante, Matthew Taylor, Hannah Hazard-Jenkins, Elena N. Pugacheva, Emidio E. Pistilli

**Affiliations:** 1 MD/PhD Medical Scientist Program, West Virginia University School of Medicine, Morgantown, WV 26506 USA; 2 Cancer Institute, West Virginia University School of Medicine, Morgantown, WV 26506 USA; 3 Division of Exercise Physiology, Department of Human Performance, West Virginia University School of Medicine, Morgantown, WV 26506 USA; 4 Genomics Core Facility, West Virginia University, Morgantown, WV 26506 USA; 5 West Virginia School of Osteopathic Medicine, Lewisburg, WV 24901 USA; 6 Department of Surgery, West Virginia University School of Medicine, Morgantown, WV 26506 USA; 7 Department of Biochemistry, West Virginia University School of Medicine, Morgantown, WV 26506 USA; 8 Department of Microbiology, Immunology, and Cell Biology, West Virginia University School of Medicine, Morgantown, WV 26506 USA; 9 West Virginia Clinical and Translational Sciences Institute, West Virginia University School of Medicine, Morgantown, WV 26506 USA

**Keywords:** Breast cancer, Cancer metabolism

## Abstract

Increased susceptibility to fatigue is a negative predictor of survival commonly experienced by women with breast cancer (BC). Here, we sought to identify molecular changes induced in human skeletal muscle by BC regardless of treatment history or tumor molecular subtype using RNA-sequencing (RNA-seq) and proteomic analyses. Mitochondrial dysfunction was apparent across all molecular subtypes, with the greatest degree of transcriptomic changes occurring in women with HER2/neu-overexpressing tumors, though muscle from patients of all subtypes exhibited similar pathway-level dysregulation. Interestingly, we found no relationship between anticancer treatments and muscle gene expression, suggesting that fatigue is a product of BC per se rather than clinical history. In vitro and in vivo experimentation confirmed the ability of BC cells to alter mitochondrial function and ATP content in muscle. These data suggest that interventions supporting muscle in the presence of BC-induced mitochondrial dysfunction may alleviate fatigue and improve the lives of women with BC.

## Introduction

Muscle dysfunction in individuals with cancer is commonly thought to be a consequence of muscle atrophy, which is a major component of the paraneoplastic syndrome known as cancer cachexia^1,2^. While studies in men with cancer support the claim that muscle functional capacity is dependent on muscle size, women with cancer report a significant degree of muscle dysfunction despite typically remaining weight-stable^3–5^. Muscle dysfunction in breast cancer (BC) commonly presents as a persistent, severe fatigue that frequently contributes to dose reduction or treatment cessation and is an independent predictor of survival in a variety of cancer types, including BC^6–11^. Thus, it is probable that improving muscle fatigue will improve both quality of life and survival in BC.

At present, there are several purported contributory factors for cancer-related fatigue, including immunological responses to tumor growth; side-effects of cancer therapies; depression and/or emotional distress; anemia; hormonal, nutritional, and metabolic disturbances; and inadequate physical activity^8,12^. Pharmacological treatments for cancer-related fatigue exhibit limited and inconsistent success, in part because determining the mechanisms contributing to fatigue in a given patient can be quite challenging, particularly in patients with early stage disease and those not receiving anticancer treatments^13^. Identifying mechanisms of BC-related fatigue that are intrinsic to skeletal muscle, generalizable across BC subtypes, and independent of treatment status could significantly aid in the development of appropriate therapies, which would be applicable to a large number of patients experiencing BC-associated fatigue.

Our laboratory has recently reported that skeletal muscle of women with BC exhibits a distinct gene expression signature that is not dependent on molecular subtype^14^. Furthermore, we have identified signaling via the metabolic regulators of the peroxisome-proliferator activated receptor (PPAR) family as potential key mediators of fatigue in women with BC and female mice-bearing BC patient-derived orthotopic xenografts (PDOXs)^15^. Our previous analyses did not include women with primary tumors that overexpressed HER2/neu in the absence of estrogen receptor (ER) and progesterone receptor (PR) expression. In the current study, we have expanded our analyses into all molecular subtypes, including both transcriptomic and proteomic analyses of muscle biopsies from patients with HER2/neu-overexpressing tumors, and significantly increased our sample size to create, to our knowledge, the largest study of transcriptomic and proteomic changes in muscle of women with BC. We tested the hypothesis that BC induces a common molecular response in skeletal muscle that is independent of the molecular subtype of the tumor and the patient’s treatment history.

## Results

### Patient characteristics

A total of 51 BC patients representing 4 breast tumor subtypes and 20 noncancer controls provided *pectoralis major muscle* biopsies and/or detailed clinical information for use in the present study. There were no differences in mean body mass index (BMI) between non-cancer controls and BC patients; average BMI in the control group was categorized as Class I Obesity and in the BC patients was categorized as Overweight.

In addition, there were no significant differences in the percent (%) change in BMI, body fat %, or lean body mass between controls and BC patients. There were no differences in BMI between any of the four breast tumor subtypes (Table 1).

**Table 1:** Patient characteristics.

Patient group	BMI (kg m^−2^)	BMI change (%)	Body fat (%)	Lean body mass (kg)	Number of days in record
Control	32.7 ± 7.8	−0.16 ± 2.9	37.7 ± 7.5	45.9 ± 6.3	220.1 ± 135.1
All cancer	29.9 ± 7.4	−0.32 ± 5.10	35.5 ± 3.9	46.4 ± 5.6	156.0 ± 242.0
ERPR	28.6 ± 4.5	−0.54 ± 2.9			84.20 ± 161.1
HER2	31.1 ± 7.8	1.12 ± 5.9			159.9 ± 131.9
TN	29.6 ± 6.5	0.07 ± 6.3			313.5 ± 426.6
TP	31.6 ± 11.6	−1.47 ± 6.5			125.8 ± 94.99
*t* Test, two-tailed *p* (Df, *t*)	*p* = 0.18 (69, 1.36)	*p* = 0.90 (69, 0.13)	*p* = 0.24 (41, 1.19)	*p* = 0.82 (41, 0.22)	*p* = 0.27 (69, 1.12)
ANOVA (Df_between_, Df_within_, *F*)	*p* = 0.70 (3, 47, 0.47)	*p* = 0.72 (3, 47, 0.45)			*p* = 0.08^*^ (3, 47, 2.38)

All values are presented as mean ± standard deviation.

*t* Test: control vs. all cancer.

ANOVA: among patient groups.

*Df* degrees of freedom, *BMI* body mass index, *ER* estrogen receptor, *PR* progesterone receptor.

*p < 0.10.

### Skeletal muscle gene expression profiles

**Fig. 1:**
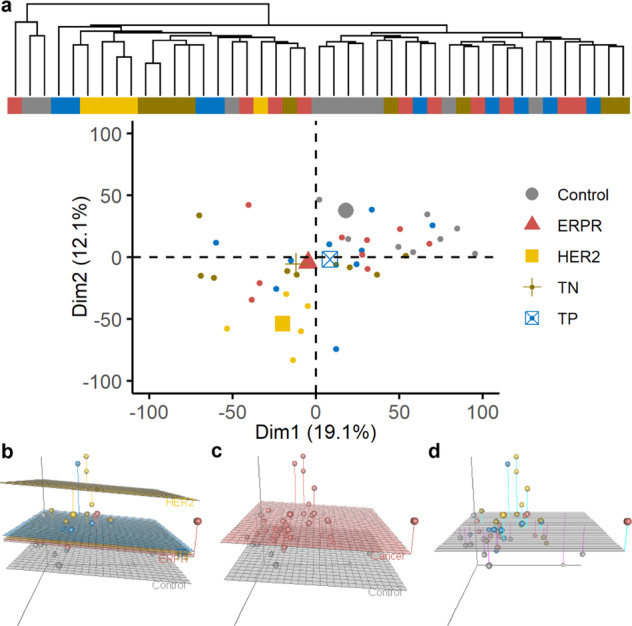
Skeletal muscle gene expression profiles. Unsupervised hierarchical clustering of individual patients on normalized, log-transformed RNA-seq gene expression data of the 10,000 most variable genes across all patients (**a** top) and first two principal components calculated using filtered, normalized, log-transformed RNA-seq gene expression data with samples colored according to BC molecular subtype with group geometric means denoted with larger symbols (**a** bottom). MDS dissimilarity matrix of overall gene expression data represented in three-dimensions, with each dot representing an individual patient. Regression planes are color-coded based on molecular subtype (**b**), binary disease status (**c**), or the null model (**d**).

Skeletal muscle biopsies from patients diagnosed with BC (*n* = 33) and non-cancer controls (*n* = 10) were used for RNA-seq, and BC patients were classified based on molecular subtype of their primary tumor, as follows: luminal (ERPR)—positive for ER and PR without overexpression of HER2/neu; HER2—overexpression of HER2/neu in the absence of ER and PR expression; triple negative (TN)—absence of ER, PR, and HER2/neu expression; and triple positive (TP)—presence of ER and PR expression, and overexpression of HER2/neu (ERPR *n* = 10, HER2 *n* = 5, TN *n* = 9, TP *n* = 9). Un-normalized gene expression counts are provided for each sample (Supplementary Data 1)^16^. Unsupervised clustering and principal component analyses suggested the possibility of clustering based on molecular subtype, particularly with regard to patients with tumors overexpressing HER2/neu in the absence of ER or PR (Fig. 1a). Multidimensional scaling (MDS) analysis restricted to three-dimensions revealed that the gene expression profiles of skeletal muscle from patients with ERPR, TP, and TN tumors were similar, while the profile from skeletal muscles from patients with HER2/neu-overexpressing tumors was significantly different (Fig. 1b). Overall fit of the MDS model was dramatically improved by the use of BC subtype as a covariate (Fig. 1b, adjusted *R*^2^ = 0.44, *p* = 0.0001) rather than a model including only binary disease status (Fig. 1c, adjusted *R*^2^ = 0.20, *p* = 0.008), which was already a significant improvement over the null model (Fig. 1d).

To address the question of whether this obvious difference in overall muscular gene expression between groups was inherent to differences in the primary tumor type or the myriad of clinical characteristics that could potentially differ between groups, we assessed the relationship between clinical characteristics and skeletal muscle gene expression in the context of a multivariate linear regression model, using the three-dimensions of the MDS dissimilarity matrix as response variables. Among the various treatment types, body composition, serum albumin, and changes in body mass, the only assessed variable to yield statistical significance at *α* = 0.05 when used as a single independent variable was patient group (i.e., Control, ERPR, HER2, TN, and TP), with serum albumin nearing statistical significance (Table 2). Notably, chemotherapy, radiotherapy, and hormonal treatments did not correlate with overall gene expression patterns, nor did the patient’s trend of weight change over time. Using forward selection, a final model including patient group and serum albumin was identified as the best-fitting model for predicting muscular gene expression from clinical data (Table 3).

**Table 2:** Possible clinical predictors of muscular gene expression.

Variable	Variable type (levels or range)	Pillai’s trace	Num Df	Den Df	*p*
Group	Factor (Control, ERPR, HER2, TN, and TP)	0.51	12	111	0.04^*^
Serum albumin	Numeric	0.20	3	29	0.08^**^
N staging	Factor (N0, N1, N1a, N2, N3)	0.52	12	84	0.15
# of Chemotherapy Txs	Numeric (0-2)	0.10	3	38	0.25
# of Radiation Txs	Numeric (0-1)	0.10	3	38	0.26
T Staging	Factor (T1, T1a, T2, T2A, T3, T4)	0.51	15	81	0.37
# of Hormonal Txs	Numeric (0-1)	0.07	3	38	0.43
LBM %	Numeric	0.12	3	19	0.45
BMI	Numeric	0.05	3	38	0.56
Ave. daily BMI change	Numeric	0.03	3	37	0.78
M staging	Factor (M0, MX)	0.01	3	29	0.95

*Num* numerator, *Den* denominator, *Df* degrees of freedom, *LBM %* lean body mass as a percentage of total body mass, *Ave* average, *T* tumor, *N* lymph node, *M* metastatic, *Txs* courses of treatments.

*p < 0.05.

**p < 0.10.

**Table 3:** Final clinical predictors of muscular gene expression.

Model formula	*F*-statistics for model fit, by dimension	Pillai’s trace (*p* value)	Wilk’s lambda (*p* value)
(D1–D3) ~Patient Group	D1: 0.3 D2: 6.9 D3: 0.4	0.506 (0.044)^*^	0.522 (0.020)^*^
(D1–D3) ~Patient Group + Serum Albumin	D1: 0.7 D2: 3.5 D3: 2.1	0.744 (0.052)^**^	0.401 (0.049)^*^

*D1–D3* individual dimensions of RNA-sequencing data as three-dimensional dissimilarity matrix.

*p < 0.05.

**p < 0.10.

**Fig. 2:**
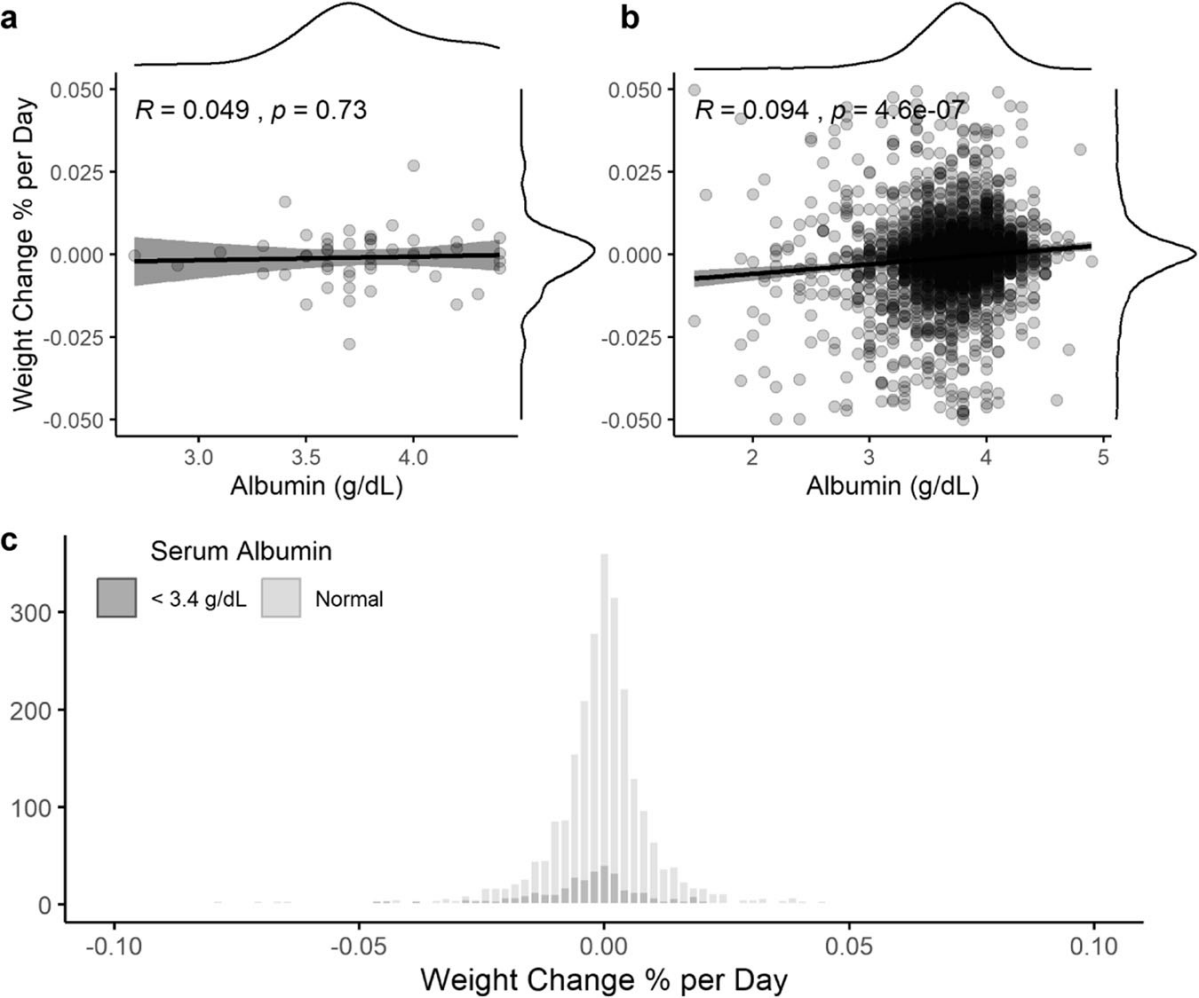
Serum albumin and weight loss. Linear regression analysis of trends in weight change predicted by serum albumin in patients with BC who provided muscle biopsies and at least one serum albumin measurement. Gray dots represent individual patients; gray shading represents 95% confidence interval of the black regression line, with marginal kernel density plots provided for both variables (**a** *n* = 51). Linear regression analysis of trends in weight change predicted by serum albumin in a retrospective chart review of patients with BC. Gray dots represent individual patients; gray shading represents 95% confidence interval of the black regression line, with marginal kernel density plots provided for both variables (**b** *n* = 3001). Histogram representing individualized rate of weight change in a retrospective chart review of patients with BC, with patients grouped by serum albumin level (**c** *n*_low_ = 442, *n*_normal_ = 2557).

Because serum albumin appeared to provide predictive value for skeletal muscle gene expression data, and because serum albumin is commonly used by oncologists to monitor patients’ nutritional status, the relationship between serum albumin and changes in BMI over time were assessed in the group of patients that provided biopsies. There was no correlation observed between serum albumin at the date of biopsy collection and the individual’s rate of weight change over time (Fig. 2a). Similar results were obtained in a retrospective chart review of 3001 patients with BC. While there was a statistically significant correlation between a patient’s first record of serum albumin and the patient’s rate of weight change in this large sample (Fig. 2b), the effect size may well be clinically insignificant (*R* = 0.094). The average daily weight change was negligible in women with normal serum albumin as well as those with low serum albumin (<3.4 g dL^−1^), with both groups having means within one standard deviation of 0 (Fig. 2c). For a patient to be considered cachectic by traditional standards, an average daily weight loss of at least 0.027% would be required to lose 5% of their weight in 6 months^1,2^. In our large cohort, a logistic regression analysis was conducted using a threshold of 3.4 g dL^−1^ serum albumin to predict whether a patient would exhibit this rate of weight change. In this analysis, omnibus model fit was significant at *ɑ* < 0.05, though effect size as determined by Nagelkerke’s pseudo-*R*^2^ was very small and indicates a very weak predictive value (*R*^2^ = 0.08). In addition, a threshold of 3.4 g dL^−1^ serum albumin was only 40% sensitive to identifying this level of weight change and only yielded a positive predictive value of 17.3%. In other words, 60% of BC patients exhibiting a rate of weight loss consistent with cachexia had normal serum albumin, while BC patients with serum albumin <3.4 g dL^−1^ only had a 17.3% chance of exhibiting a rate of weight change consistent with cachexia.

### Differential gene expression analysis by subtype

**Fig. 3:**
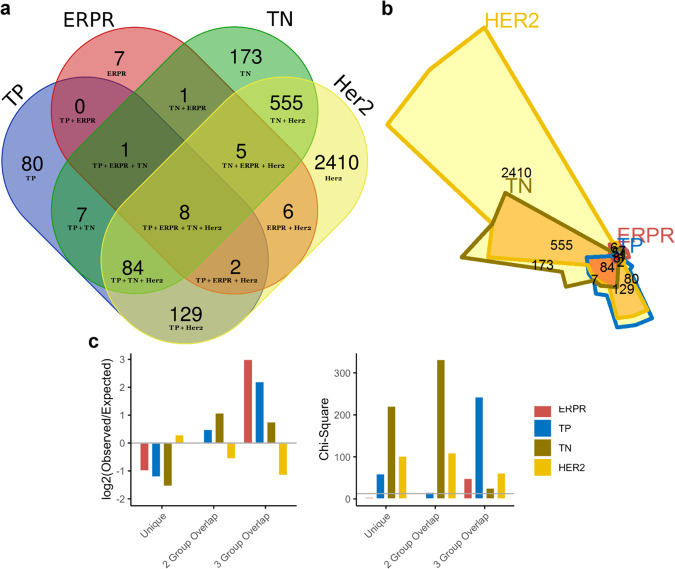
Differential gene expression analysis by subtype. Venn diagram representing DEGs in muscle in each molecular subtype (**a**) and corresponding area-representative Chow–Ruskey diagram (**b**). Chi-square analysis of the number of DEGs uniquely differentially expressed in each subtype, the number of DEGs involved in two- and three-way overlaps among subtypes, showing the log-fold change between observed and expected numbers in each category. The chi-square critical value of 12.592 (6 degrees of freedom) is denoted on the chi-square *y*-axis by a gray horizontal line. A chi-square value larger than 12.592 is statistically significant at *α* = 0.05 (**c**).

Differentially expressed genes (DEGs) within skeletal muscle were first identified by comparing BC patients by subtype to control. Considerable overlap of DEGs was observed between the four breast tumor subtypes. Of the 3468 genes identified as differentially expressed in at least one subtype, only 7 (0.2%) were unique to ERPR patients, 173 (5.0%) to TN patients, and 80 (2.3%) to TP patients. However, 2410 genes (70%) were unique to HER2 patients, and only 8 (0.23%) were differentially expressed in all patient groups (Fig. 3a, b). These observations were quantified and reveal that there was an approximately twofold fewer-than-expected number of unique DEGs in muscle of patients with ERPR, TP, and TN tumors if the DEGs were independent of subtype. Specifically, one would expect 14, 186, and 508 unique DEGs in each subtype, respectively, whereas we actually observed 7, 80, and 173 DEGs in these groups. Further, HER2 patients’ muscle exhibited twofold fewer-than-expected DEGs shared with any combination of two other subtypes (observed = 5 + 2 + 84 = 91; expected total = 203; overall *χ*^2^ on 6 degrees of freedom = 1224, *p* = 3 × 10^−261^). This indicates that the ERPR, TP, and TN groups share a greater number of DEGs in skeletal muscle than one would expect if the DEGs were independent of subtype and in contrast, muscle from HER2 patients does not exhibit the same similarity to the other subtypes in terms of shared DEGs (Fig. 3c). Collectively, these data demonstrate that transcriptional responses in skeletal muscle of patients with ERPR, TP, and TN tumors are highly similar, in support of previous data from our laboratory^14,15^. Furthermore, the transcriptional responses in muscles from patients with HER2/neu-overexpressing tumors partially overlap with the other subtypes, but exhibit a significant contrast to the other three subtypes, suggesting that this tumor type is associated with a unique transcriptional adaptation within skeletal muscle.

### Pathway analysis by subtype

**Fig. 4:**
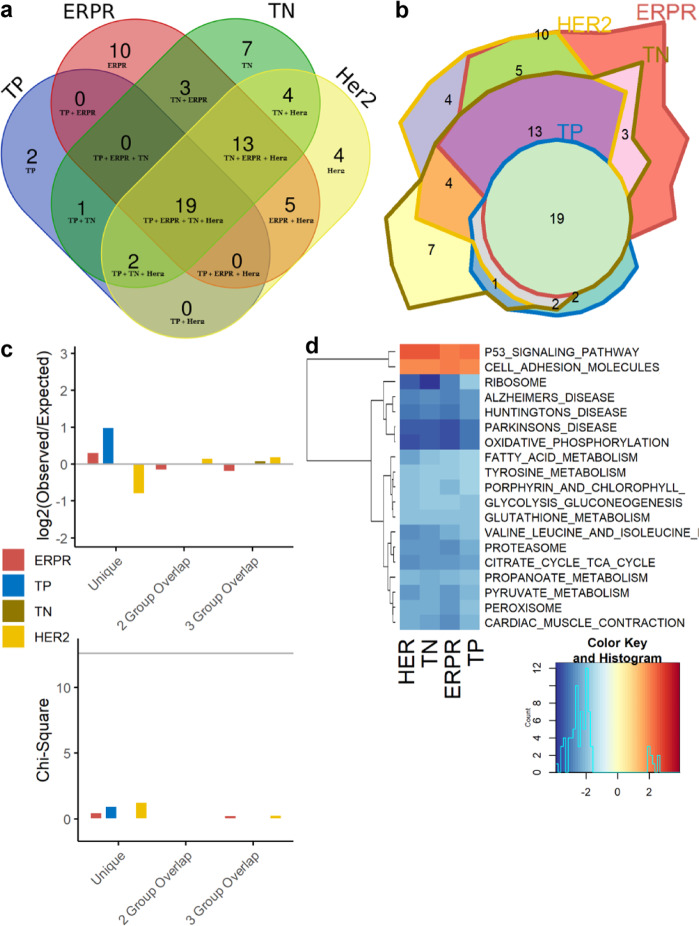
Dysregulated pathway analysis by subtype. Venn diagram representing significantly dysregulated KEGG pathways in muscle in each molecular subtype (**a**) and corresponding area-representative Chow–Ruskey diagram (**b**). Chi-square analysis of the number of pathways uniquely dysregulated in each subtype and those involved in two- and three-way overlaps between subtypes, showing the log-fold change between observed and expected numbers in each category. The chi-square critical value of 12.592 (6 degrees of freedom) is denoted on the chi-square *y*-axis by a gray horizontal line. A chi-square value larger than 12.592 is statistically significant at *α* = 0.05 (**c**). Heatmap of normalized enrichment scores from GSEA for the 19 commonly dysregulated pathways across all subtypes, with the four columns of the heatmap representing the four molecular subtypes. Lower enrichment scores in blue indicate genes within that set are generally downregulated in the BC group, and higher enrichment scores in red indicate general upregulation in BC group relative to control (**d**).

The Broad Institute’s Gene Set Enrichment Analysis (GSEA) tool^17^ was used to infer pathway-level dysregulation based on trends in transcriptomic changes, assessing all pathways in the Kyoto Encyclopedia of Genes and Genomes (KEGG)^18^. Identified pathways were shared between all breast tumor subtypes to a greater extent than individual genes. Of the 70 pathways identified as dysregulated in any subtype, 19 (27.1%) pathways were identified as significantly dysregulated in all 4 breast tumor subtypes relative to control. Two (2.9%) pathways were uniquely dysregulated in muscle from patients with TP tumors, 10 (14.2%) in patients with ERPR tumors, 7 (10.0%) unique to TN patients, and 4 (5.7%) uniquely dysregulated in patients with HER2/neu-overexpressing tumors (Fig. 4a–c). Chi-square analysis to test whether the number of pathways shared between subtypes differed between groups was nonsignificant (overall *χ*^2^ on 6 degrees of freedom = 3.67, *p* = 0.72), indicating that the four BC subtypes share a common core of dysregulated pathways (Fig. 4c). The 19 overlapping pathways clearly show a substantial degree of metabolic alteration, including pathways involved in carbohydrate, lipid, and protein metabolism (Fig. 4d). While the enrichment of Alzheimer’s, Parkinson’s, and Huntington’s diseases may seem unusual, these pathways include a large number of mitochondrial genes, explaining why these pathways cluster with oxidative phosphorylation. The enrichment of the KEGG Peroxisome pathway is significant, as our laboratory has previously identified the PPAR proteins as likely upstream regulators of muscle fatigue in BC^14,15^. Though our multivariate linear regression model did not show neoadjuvant therapies to predict overall gene expression, the upregulation of transcripts in the KEGG p53 signaling pathway is likely a response to chemotherapy and/or radiotherapy.

### HER2 patient proteomics

**Fig. 5:**
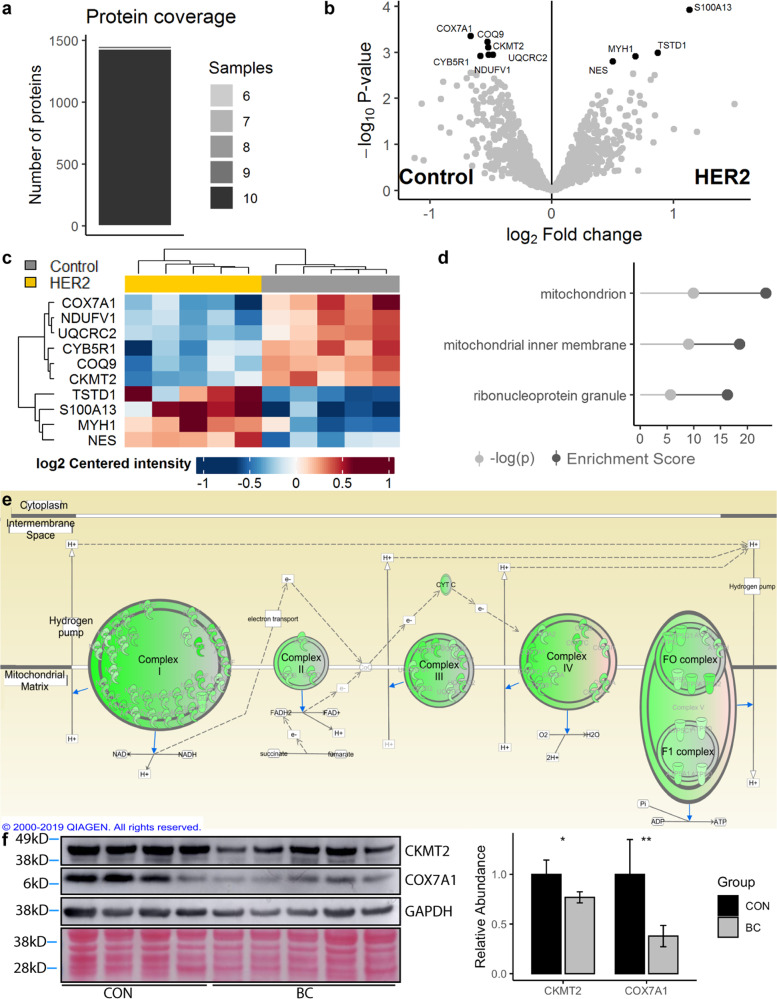
HER2 patient proteomics. Stacked bar chart representing the number of unique proteins detected by the number of samples (**a** *n* = 10 biopsies, *n* = 1555 unique proteins). Differential expression analysis of detected proteins, represented as a volcano plot with significantly differentially expressed proteins (DEPs) identified in black (**b**). Heatmap of DEPs, with the ten columns representing the ten samples assayed (**c**). Enrichment analysis on the DEPs, querying Gene Ontology 2018 Cellular Components via Enrichr; top three results as ranked by enrichment score (**d**). Ingenuity Pathway Analysis representation of the mitochondrial electron transport chain, with proteins detected at lower abundance in the BC patient group notated in green (**e**). Western blot validation of two DEPs, using an independent cohort of BC and CON patients with Ponceau staining and GAPDH presented as loading controls. Quantification was completed using two blots conducted in parallel as technical replicates, with a single representative blot presented (**f**).

Because the HER2 patient group exhibited the greatest degree of transcriptomic dysregulation, muscle biopsies from these patients (*n* = 5) were selected for proteomic analysis and compared to control surgical patients (*n* = 5). Un-normalized protein abundance estimates are provided for each sample (Supplementary Data 2)^16^. Scaled, log-transformed expression data from proteomic analyses were correlated with scaled, log-transformed RNA-seq expression data in a gene-wise fashion. Totally, 8/8 individuals with matched RNA-seq and proteomic analyses were found to have moderately strong correlation (Pearson’s *R* range 0.48–0.52, Supplementary Fig. 1). A total of 1555 unique proteins were detected across all samples, 1259 of those at a high confidence level, with most proteins being detected in all 10 samples (Fig. 5a). Differential expression analysis detected only a small number of significantly differentially expressed proteins (DEPs) (FDR < 0.05, Fig. 5b), though there are obvious physiological implications in the small set. The six downregulated DEPs were all identified as mitochondrial components (Fig. 5c), representing a statistically significant enrichment of mitochondrial components in this small set of DEPs (Fig. 5d, *p* = 3.2 × 10^−6^). Expanding our analysis to both significant DEPs and insignificant trends in protein expression, a strong signal for aberrant mitochondrial function was once again detected. Nearly every protein involved in the mitochondrial electron transport chain was quantified as having a lower level of protein expression in the HER2 patients relative to controls (Fig. 5e, Supplementary Data 3^16^), including proteins encoded by both nuclear DNA and mitochondrial DNA. GSEA results from the proteomic data were highly similar to those obtained in the RNA-seq analysis (Supplementary Fig. 2), with significantly affected pathways, including oxidative phosphorylation (NES = −2.97, FDR < 0.001), citrate cycle (NES = −2.12, FDR < 0.001), peroxisome (NES = −1.71, FDR = 0.02), and fatty acid metabolism (NES = −1.66, FDR = 0.27), among other pathways. Two mitochondrial proteins identified as significantly differentially expressed in the HER2 patient group were selected for validation by Western blotting in a completely independent cohort of patients, without regard to patient molecular subtype. Both proteins assessed were confirmed to have lower abundance in the BC group compared to control (Fig. 5f).

### Subtype-independent DEG analysis and experimental validation

**Fig. 6:**
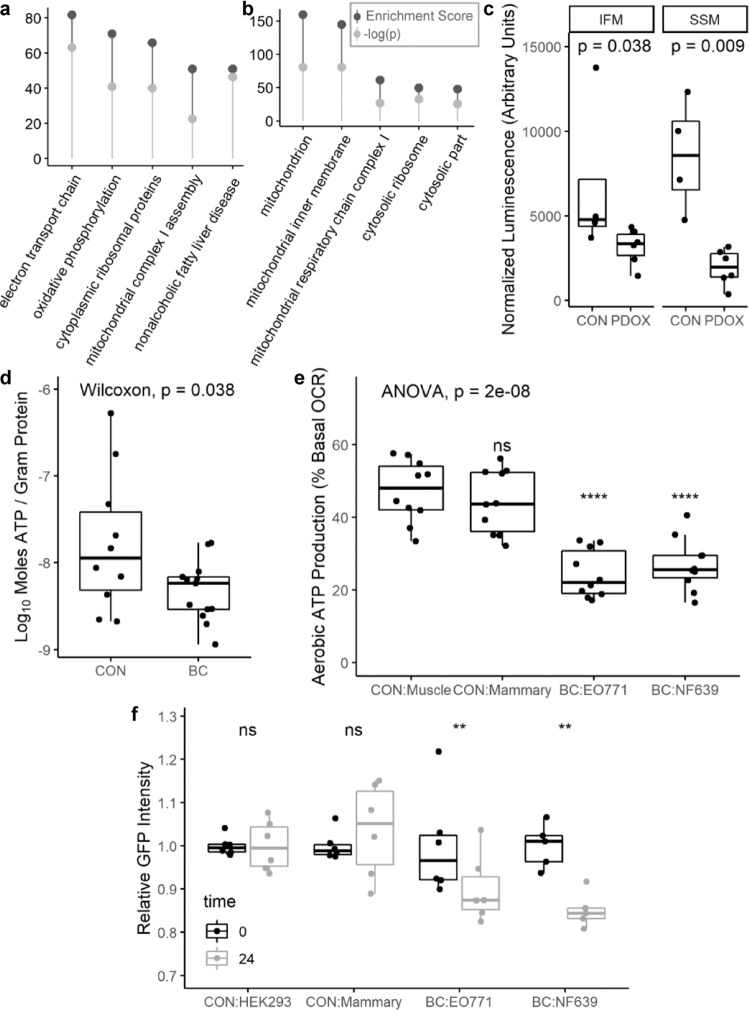
Subtype-independent DEG analysis and experimental validation. Enrichment analysis of DEGs, querying WikiPathways 2019 Human database. Top hits ranked by enrichment score (**a**). Enrichment analysis of DEGs, querying Gene Ontology 2018. Top hits ranked by enrichment score (**b**). Enrichment score and *p* value reported as provided by Enrichr. A −log(*p*) > 1.3 corresponds to *p* < 0.05 (**a**, **b**). ATP content in *quadriceps muscle* of control (*n* = 4) and PDOX-bearing (*n* = 6) mice as assessed by chemiluminescence in two mitochondrial subpopulations. ATP content in the PDOX mitochondria was compared to control using the Wilcoxon rank sum test (**c**). ATP content in *pectoralis major muscle* of control (*n* = 10) and BC patients (*n* = 13) as assessed by chemiluminescence using a standard curve for absolute quantification and normalized to protein content. ATP content in the BC group was compared to control using the Wilcoxon rank sum test (**d**). Aerobic ATP production as a percentage of basal oxygen consumption rate (OCR) in differentiated C2C12 myotubes treated for 48 h with media conditioned by C2C12 (CON-Muscle), EpH4-EV (CON-Mammary), EO771 (BC), or NF639 (BC) cells (*n* = 10 per group). Overall significance determined by one-way ANOVA followed by two-tailed Student’s *t* tests with Holm–Bonferroni correction comparing each treatment group to CON-Muscle (**e**). Normalized GFP intensity in HEK293-PPRE-H2b-eGFP reporter cells at baseline and after treatment with conditioned media for 24 h (*n* = 6 for HEK293, EpH4-EV, and EO771; *n* = 5 for NF639). Normalized GFP intensity values at 24 h were compared to baseline measurements using paired samples two-tailed *t* tests with Holm–Bonferroni correction of *p* values (*p*. adj) (**f**). The width of the box represents the interquartile range (IQR) and whiskers extend to the single extreme measurement in both directions, unless the extreme measurement is considered an outlier, in which case the extreme value is represented as a dot without a connected whisker. Median values are represented by the horizontal line through each boxplot. Outliers in this context are defined as values more extreme than 1.5 × IQR.

To cross-validate our RNA-seq GSEA results using an independent software, pathway-level enrichment analysis was conducted using the list of genes that were significantly differentially expressed when comparing all BC patients to control (FDR < 0.10). In this analysis, we observed a strong signal indicating aberrant mitochondrial function across multiple databases queried. For example, three of the top four enriched pathways identified when querying the WikiPathways 2019 Human database, included electron transport chain system in mitochondria (WP111), oxidative phosphorylation (WP623), and mitochondrial complex I assembly (WP477, Fig. 6a); and the three most enriched cellular components from the 2018 Gene Ontology project were all mitochondrial components (Fig. 6b).

To test this prediction of altered mitochondrial function in tissues distant from the primary tumor in vivo, we generated six female BC-PDOX mice and isolated live mitochondria from their skeletal muscle at euthanasia for quantification of ATP content. We found a significant reduction in ATP content within both of the two major skeletal muscle mitochondrial subpopulations, the interfibrillar mitochondria (IFM) and subsarcolemmal mitochondria (SSM), relative to female control animals (Fig. 6c). We additionally quantified ATP content in snap-frozen muscle biopsies from a large group of women with BC and control patients. Consistent with our in silico predictions and murine studies, bulk muscle from BC patients was quantified as having significantly less ATP per gram of protein than control muscle, though the large degree of variability in absolute quantifications should be noted (Fig. 6d). In vitro assays were conducted to determine whether mitochondrial dysfunction in muscle is a direct response to BC-secreted factors or an indirect response mediated by other tissues. In support of a direct response, conditioned media from the luminal EO771 BC cell line and the HER2/neu overexpressing NF639 line significantly repressed aerobic ATP production in differentiated C2C12 myotubes, whereas media conditioned by either the normal mammary epithelial cell line EpH4-EV or C2C12 myoblasts did not (Fig. 6e). To validate the role of the PPAR signaling proteins in mediating the systemic response to BC-secreted factors, conditioned media was isolated from EO771 and NF639 cells and applied to HEK293 cells stably expressing a PPAR-responsive promoter driving GFP expression. Media conditioned by both BC cell lines significantly repressed GFP intensity relative to reporter cell conditioned media, while media conditioned by a normal mammary epithelial cell line did not alter GFP signal relative to control (Fig. 6f). These data indicate that BC cells secrete a substance that is capable of directly influencing metabolic function in skeletal muscle, which may be related to a repression of PPAR-mediated transcriptional activity.

## Discussion

BC-induced muscle dysfunction is a common problem of unclear etiology with few therapeutic options. Here, we sought to identify possible mechanisms of fatigue that are generalizable across BC subtypes and are independent of treatment status by assessing statistical relationships between patients’ clinical characteristics and overall skeletal muscle molecular composition.

In support of our laboratory’s previous publications^14,15^, we found that women with three molecular subtypes of BC, those being ERPR, TN, and TP BC, exhibit overall similarity in muscular gene expression. Remarkably, patients with tumors overexpressing HER2/neu in the absence of ER and PR expression exhibited a markedly different muscular gene expression profile. However, gene expression data from all subtypes pointed to similar pathway-level dysregulation, indicating that the mechanisms leading to muscle fatigue in BC patients may indeed be generalizable across subtypes, although we acknowledge this conclusion is based on a limited sample size across individual BC subtypes. In assessing the commonalities across patients, we observed significant dysregulation of metabolic pathways in all groups of BC patients, and proteomic analysis of patients with HER2/neu-overexpressing tumors showed decreased abundance of nearly all proteins involved in the mitochondrial electron transport chain. Because mitochondrial density in skeletal muscle has been shown to correlate with abundance of ETC complex proteins and skeletal muscle oxidative capacity^19^, it is likely that BC patients also have decreased mitochondrial density in their skeletal muscle as well as decreased oxidative capacity. We propose then that BC-secreted factors induce muscle dysfunction by abrogating oxidative capacity via alteration of mitochondrial biogenesis, mitophagy, or fission/fusion dynamics. Both in vitro and in vivo assays confirm that factors from BC cells alter skeletal muscle ATP content and/or aerobic ATP production, perhaps via dysregulation of the PPAR-signaling pathway.

Our laboratory’s interest in the PPAR family of proteins arose from our previous RNA-seq analysis of muscle from BC patients and PDOX-bearing mice, which our group found to recapitulate the clinical phenotype of increased muscle fatigue without muscle atrophy or bodyweight loss^15^. These proteins have demonstrated roles in whole-body energy regulation, are critical regulators of mitochondrial function in multiple tissues, and are targets of multiple FDA-approved agents in the treatment of type 2 diabetes and hyperlipidemia. Among the three PPAR isoforms, we identified PPARG as a key regulator in BC-induced muscle dysfunction observed in PDOX mice^15^. PPARG is a ligand-activated nuclear receptor that, upon activation by a variety of endogenous and synthetic lipids, forms complexes with retinoid X receptor and cofactors such as the peroxisome proliferator-activated receptor-γ coactivator 1α (PGC1α), and stimulates transcription of downstream genes. This relationship with PGC1α is particularly relevant, as PGC1α is known to be a master regulator of mitochondrial biogenesis in several tissues, including skeletal muscle^20^. PGC1α also participates in the regulation of other metabolic processes, including gluconeogenesis, muscle fiber-type specification, and control of antioxidant expression^21–23^. Therefore, the interaction between PPARG and PGC1α clearly has the potential to influence muscle function through mitochondrial mechanisms. In addition to the potential development of muscle-intrinsic mitochondrial dysfunction, a systemic consequence of dysregulated PPARG is the development of insulin resistance (IR)^24^, which is commonly associated with type 2 diabetes, obesity, and metabolic syndrome, and appears to have a bidirectional relationship with BC, with individuals with IR at greater risk of BC and BC survivors at an increased risk of IR^25,26^. We propose that the development of mitochondrial dysfunction and IR, secondary to muscular PPARG downregulation by BC, creates an environment that facilitates the development of muscle fatigue through several mechanisms, including decreased mitochondrial ATP production as well as dysregulated glucose and lipid metabolism. Pharmacological restoration of PPARG function results in the induction of a number of genes involved in insulin signaling, as well as glucose and lipid metabolism, and PPAR agonist drugs including the thiazolidinediones have shown a remarkable ability to restore insulin sensitivity in insulin-resistant conditions. Because our predictions have implicated many related metabolic pathways, we hypothesize that the development of BC induced muscle fatigue constitutes a pathology similar to type 2 diabetes/metabolic syndrome. If repression of PPAR signaling is indeed central to BC-induced muscle dysfunction, the numerous FDA-approved PPAR-agonizts could address this unmet need in clinical oncology.

A particularly surprising result in the present study was that no clinical data aside from BC molecular subtype exhibited significant correlation with skeletal muscle gene expression, including TNM staging and history of chemotherapy, radiation, or immunotherapy. In addition, patients in the HER2 group exhibited a decreased abundance of mitochondrial proteins in their muscle tissue, and importantly, we observed these responses consistently across all five biopsies from this patient group despite significant differences in anti-cancer treatment history. At the date of biopsy collection, one patient was entirely treatment naïve, one patient had completed chemotherapy for BC 10 years prior, and the remaining 3 patients received different multi-agent neoadjuvant chemotherapy in the months prior to surgery. Therefore, we propose that changes in skeletal muscle physiology seen in BC are due to tumor-derived factors rather than side effects of therapies or other patient-specific factors. This hypothesis is supported by our in vitro conditioned media experiments where tumor-derived factors directly repressed mitochondrial respiratory capacity in differentiated muscle cells. In addition, neither weight loss nor body composition were predictive of skeletal muscle gene expression, suggesting that gene expression changes observed in BC patients are unrelated to muscle atrophy or cachexia and may instead be reflective of muscle dysfunction. Therefore, the molecular alterations we highlight suggest that skeletal muscle is responding at the early stages of breast tumor growth, at a time when patients would be considered “pre-cachectic”, based on Fearon’s cachexia continuum^1^. Clinicians should be aware that patients with BC may not have dramatic changes in body weight and/or muscle mass during tumor growth or tumor-directed therapies. However, this does not necessarily imply that muscle dysfunction, in the form of persistent fatigue, is not adversely affecting patients’ quality of life.

Because albumin is often used by physicians as a measure of patients’ nutritional status and mortality risk^27–30^, we assessed serum albumin in our patient cohort and assessed its relationship to skeletal muscle gene expression and weight change. In our analysis, serum albumin was not found to be predictive of weight change, and in a larger sample was found to be only weakly predictive of cachexia risk. Yet, it was unexpectedly predictive of skeletal muscle gene expression. This indicates that serum albumin may be a useful biomarker of muscle function during pre-cachexia, a possibility supported by previous literature connecting serum albumin with muscle strength^31^ and IR^32^ in other clinical contexts. Prospective studies directly addressing the relationship between skeletal muscle function, gene expression, and serum albumin would be required to validate the clinical utility of serum albumin in identifying those at risk of cancer-induced muscle dysfunction.

In line with our previous publications^14,15^, we report that transcriptional responses to the ERPR, TP, and TN subtypes of BC are similar in terms of skeletal muscle gene expression, while muscle biopsies from patients with tumors overexpressing HER2/neu in the absence of ER and PR exhibit an unusual degree of uniqueness in terms of gene expression. We identified a strong signal for BC-induced mitochondrial dysfunction in BC patients, PDOX-bearing animals, and in in vitro assays, and proteomic analysis showed decreased protein abundance of nearly all components of the mitochondrial electron transport chain in muscle biopsies from patients with HER2/neu-overexpressing tumors relative to control. Further, we found no relationship between various BC-related treatments (surgery, chemotherapy, and radiotherapy) and changes in skeletal muscle gene expression. However, serum albumin was predictive of skeletal muscle gene expression without being predictive of weight loss, suggesting that serum albumin may be a useful indicator of BC-induced skeletal muscle dysfunction. Overall, these data indicate that BC is associated with dysfunction in mitochondrial respiration in skeletal muscle independent of molecular subtype, and this effect appears to be independent of anticancer treatments. These findings call for prospective studies assessing interventions to support skeletal muscle function in the presence of BC-induced mitochondrial dysfunction.

## Methods

### Patient information

Conduct of research involving human patients at West Virginia University is guided by the principles set forth in the Ethical Principles and Guidelines for the Protection of Human Subjects of Research (Belmont Report) and is performed in accordance with the Department of Health and Human Services policy and regulations. The procedures in this study were reviewed and approved by the WVU Institutional Review Board (IRB). A total of 71 female surgical patients provided informed consent for inclusion in this study (control *n* = 20; BC *n* = 51). Informed written consent was obtained from each subject or each subject’s guardian. Individuals could be included in this study if they (1) were deemed to have operable disease or were undergoing a diagnostic biopsy, (2) had been diagnosed with, or were suspected to have, invasive adenocarcinoma of the breast, (3) were over 21 years old, and (4) provided informed consent. Women with BC provided muscle biopsies from the *pectoralis major muscle* intraoperatively at the time of mastectomy, and control patients provided *pectoralis major muscle* samples intraoperatively during other breast surgeries. Women with BC were classified into four molecular subtypes based on immunohistochemical staining of their primary tumors: ERPR (*n* = 20), HER2 (*n* = 9), TN (*n* = 11), or TP (*n* = 11).

Information on BMI at multiple time points was collected in 12 control and 50 BC patients. The mean number of days a consented patient was followed was 170 ± 214 (control median = 170, ERPR median = 36, HER2 median = 146, TP median = 140, TN median = 180). Detailed body composition analyses were acquired in 8 control and 35 BC patients using a bioelectrical impedance scale (Tanita: model SC-240). Serum albumin levels were acquired in 3 control and 49 BC patients (ERPR *n* = 18; HER2 *n* = 9; TP *n* = 11; TN *n* = 10). For RNA-Seq analyses, biopsies were used from 10 control and 33 BC patients (ERPR *n* = 10; HER2 *n* = 5; TP *n* = 9; TN *n* = 9).

In a separate analysis of de-identified electronic medical records (EMRs) from female BC patients, body mass, and standing height data were acquired for 5201 individuals for calculation of BMI. Within this population, final analyses were completed in 3001 patients having at least two measurements for weight in addition to at least one record for serum albumin.

### RNA sequencing

*Pectoralis major muscle* biopsies were acquired intraoperatively and stored in RNA stabilization reagent (Invitrogen, Thermo Fisher, San Jose, CA) overnight at 4 °C and then at 80 °C until processing. RNA was isolated, assessed for quality, and utilized to construct libraries for RNA-Seq as previously reported^15^. Completed libraries were sequenced on one lane of the HiSeq 1500 with PE50 bp reads, with approximately 15 million reads per sample. Subsequently, Salmon was used for transcript-level abundance estimation, with both gcBias and seqBias set, and libType A^33^, using the GRCh38 genome assembly. Between 5 and 10% of reads in each sample were unable to be mapped and were excluded from analyses reported here. Transcript-level abundance estimates were summarized to the gene-level using *tximport*^34^. The resulting counts matrix was scaled to library size using *edgeR*^35^ and filtered to remove genes without a counts-per-million (CPM) value > 1 in 3 or more samples. Log-transformed CPM values for the 10,000 genes with highest variance were used as input for hierarchical clustering using *gplots*^36^. CPM values of all genes with CPM > 1 in at least three samples were scaled and log-transformed prior to distance matrix computation, which was then input for classical MDS with k = 3 and visualized in three-dimensions using *car*^37^. This three-dimensional distance matrix (3D-MDS) was then utilized as three response variables representing gene expression in the multivariate regression analysis to assess statistical relationships between clinical parameters and overall gene expression. Differential gene expression analysis was conducted using *DESeq2*^38^. Both the multivariate regression analysis and differential expression analysis are further described below, within “*RNA-Seq and clinical correlates*” in the “Statistical Analyses” section. Power was determined post hoc to be 88% using *RnaSeqSampleSize*^39^ with 1000 repetitions, *f* = 0.1, and rho = 2.

### Proteomics

#### Sample preparation

*Pectoralis major muscle* biopsies from *n* = 5 female patients with HER2/neu-overexpressing BC and *n* = 5 control breast surgical patients were acquired intraoperatively and stored in RNA-later at −80 °C until processing^40^.

Muscle biopsy samples weighing approximately 50 mg were sent on dry ice to the mass spectrometry (MS) and Proteomics Resource Laboratory at Harvard University and processed according to established protocols^41,42^. Briefly, biopsies were lysed in Covaris^®^ microTUBE-15 (Woburn, MA) microtubes with Covaris^®^ TPP buffer, using the Covaris S220 Focused-ultrasonicator instrument with 125 W power over 180 s with 10% max peak power. Samples were then chloroform/methanol precipitated, filtered, reduced, alkylated, and finally digested overnight at 38 °C in a solution containing triethylammonium bicarbonate and Promega^®^ Sequencing Grade Trypsin. Digested peptides were then incubated with tandem mass tags, using different tags for each sample.

#### LC–MS/MS

The ten samples were pooled in equal amounts and fractionated into ten fractions. Liquid chromatography (LC)–MS/MS was performed on an Orbitrap Lumos (Thermo Fisher) equipped with EASYLC1000 (Thermo Fisher). Peptides were separated onto a 100 µm inner diameter microcapillary column packed first with C18 Reprosil resin (5 µm, 100 Å, Dr. Maisch GmbH, Germany) followed by analytical column of Reprosil resin (1.8 µm, 200 Å, Dr. Maisch GmbH, Germany). Separation was achieved through applying a gradient from 5 to 27% acetonitrile in 0.1% formic acid over 90 min at 200 nl min^−1^. Electrospray ionization was enabled through applying a voltage of 1.8 kV using a homemade electrode junction at the end of the microcapillary column and sprayed from fused silica pico tips (New Objective, MA). The LTQ Orbitrap Lumos was operated in data-dependent mode for the mass spectrometry methods. The mass spectrometry survey scan was performed in the Orbitrap in the range of 395–1800 *m*/*z* at a resolution of 6 × 10^4^, followed by the selection of the 20 most intense ions (TOP20) for collision-induced dissociation (CID) in the Ion trap using a precursor isolation width window of 2 *m*/*z*, AGC setting of 10,000, and a maximum ion accumulation of 200 ms. Singly charged ion species were not subjected to CID fragmentation. Normalized collision energy was set to 35 V and an activation time of 10 ms. Ions in a 10 ppm *m*/*z* window around ions selected for MS2 were excluded from further selection for fragmentation for 60 s. The same TOP20 ions were subjected to higher-energy collisional dissociation (HCD) MS2 event in Orbitrap part of the instrument. The fragment ion isolation width was set to 0.7 m/z, AGC was set to 50,000, the maximum ion time was 200 ms, normalized collision energy was set to 27 V and an activation time of 1 ms for each HCD MS2 scan.

#### MS analysis

Raw data were submitted for analysis in Proteome Discoverer 2.2 (Thermo Scientific). Assignment of MS/MS spectra was performed using the Sequest HT algorithm by searching the data against a protein sequence database including all entries from Uniprot_Human2016_SPonly database as well as other known contaminants such as human keratins and common lab contaminants. Sequest HT searches were performed using a 20 ppm precursor ion tolerance and requiring each peptides N-/C termini to adhere with trypsin protease specificity, while allowing up to two missed cleavages. Six-plex TMT tags on peptide N termini and lysine residues (+229.162932 Da) were set as static modifications while methionine oxidation (+15.99492 Da) was set as a variable modification. An MS2 spectra assignment false-discovery rate (FDR) of 1% on both protein and peptide level was achieved by applying the target-decoy database search. Filtering was performed using Percolator^43^ (64-bit version). For quantification, a 0.02 *m*/*z* window centered on the theoretical *m*/*z* value of each the six reporter ions and the intensity of the signal closest to the theoretical *m*/*z* value was recorded. Reporter ion intensities were exported using Proteome Discoverer 2.2.

### Western blotting analysis

*Pectoralis major muscle* biopsies from *n* = 13 female patients with BC and *n* = 10 control breast surgical patients were snap-frozen and stored at −80 °C until processing. Biopsies from *n* = 7 female patients with BC and *n* = 7 control breast surgical patients were selected from this pool using stratified random sampling by patient group (i.e., BC or CON) without regard to patient molecular subtype. Protein homogenates were made using a 5 mL Wheaton tissue grinder (DWK Life Sciences Inc., Millville, NJ, USA) in a solution of 20 mM Tris HCl (pH = 7.4), 150 mM NaF, 1 mM EDTA, 1% Triton X-100, and 10% glycerol. Homogenates were cleared via brief centrifugation, and protein concentration was quantified using DC^™^ Protein Assay (Bio-Rad, CA, USA) according to manufacturer’s protocol. Totally, 12 µg of total protein was loaded per well and resolved in NuPAGE Novex 4–12% Bis–Tris Gels (ThermoFisher Scientific). Proteins were transferred to nitrocellulose membrane, Ponceau stained to assess loading, destained, blocked in 1× tris-buffered saline (TBS), 0.1% Tween20, 5% milk, then incubated with primary antibody overnight at 4 °C. Membranes were then washed prior to application of appropriate secondary antibody (ThermoFisher Scientific), and again prior to application of Pierce ECL Western Blotting Substrate (ThermoFisher Scientific). Relative band intensity was quantified using the GE Amersham Imager 600 (GE Healthcare Life Sciences, Marlborough, MA, USA). Quantification was completed using two blots conducted in parallel as technical replicates. GAPDH and Ponceau staining are presented as loading controls but were not used to normalize densitometry data. Primary antibodies were diluted 1:1000 in 1× TBS, 0.1% Tween 20 and 5% milk, and included CKMT2 (ThermoFisher Scientific, #PA5-28591), COX7A1 (ThermoFisher Scientific, #PA5-67696), and GAPDH (Cell Signaling Technology, Massachusetts, USA, #2118S). Full uncropped blot images are presented in Supplementary Fig. 3.

### BC patient muscle ATP quantification

*Pectoralis major muscle* biopsies from *n* = 13 female patients with BC and *n* = 10 control breast surgical patients were snap-frozen and stored at −80 °C until processing as described under “Western blotting analysis.” All available snap-frozen muscle biopsies were included in analysis of ATP content. ATP content was quantified using the ENLITEN^®^ ATP Assay System Bioluminescence Detection Kit (Promega, Wisconsin, USA) according to manufacturer’s protocol, using the manufacturer’s recommended standard curve for calculation of absolute ATP content from luminescence intensity and The FlexStation^®^ 3 Multi-Mode Microplate Reader (Molecular Devices^®^, CA, USA). ATP content was normalized to protein concentration, quantified using the DC^™^ Protein Assay (Bio-Rad, CA, USA).

### BC-PDOX skeletal muscle mitochondria metabolic analysis

Animal experiments were approved by the WVU Institutional Animal Care and Use Committee, and conducted in accordance with the Guidelines for Ethical Conduct in the Care and Use of Nonhuman Animals in Research. BC-PDOX mice were created by implanting human BC tumor fragments into the mammary fat pad of female NOD.CG-Prkdscid Il2rgtm1 Wjl/SzJ/0557 (NSG) mice (*n* = 6), as described previously^15^. PDOX-bearing animals were euthanized approximately 30 days after reaching a tumor volume of 200 mm^3^. Control female NSG mice of similar age (*n* = 4) were euthanized at the same time as tumor-bearing animals and tissues were processed identically in both groups. Immediately after death, both quadriceps muscles from each mouse were quickly removed and IFM and SSM mitochondria were isolated separately according to previously described methods^44–47^, combining the two muscles to obtain sufficient tissue for downstream applications. Mitochondrial isolates were stored at −80 °C until analysis. ATP content was quantified in each mitochondrial subpopulation using the ENLITEN^®^ ATP Assay System Bioluminescence Detection Kit (Promega) according to manufacturer’s protocol with minor modifications, as follows. Mitochondrial isolates were lysed in 1% trichloroacetic acid for 5 min then diluted 1:10 in 0.1 mol L^−1^ tris base (pH = 7.8). The resulting samples were loaded into a black-walled microplate, in duplicate, and mixed 1:1 with luciferase/luciferin reagent. Luminescence intensity was immediately read using the FlexStation^®^ 3 Multi-Mode Microplate Reader (Molecular Devices^®^, CA, USA). Luminescence intensity values were blank-corrected and normalized to account for differing protein concentrations, which were quantified using the DC^™^ Protein Assay (Bio-Rad, CA, USA).

### Cell culture

All cell lines were obtained from ATCC (Virginia, USA) with the exception of EO771 cells, which were obtained through a Material Transfer Agreement with Wake Forest University. Cells were cultured in Gibco DMEM (Thermo Fisher, MA, USA) supplemented with 10% heat inactivated fetal bovine serum (Atlanta Biologicals, GA, USA) and Gibco penicillin/streptomycin (Thermo Fisher) at 37 °C with 6% CO_2_. Cell lines utilized include EpH4-EV (immortalized normal murine mammary epithelium), EO771 (murine luminal BC), NF639 (murine HER2/neu-overexpressing BC), HEK293 (human embryonic kidney), and C2C12 (murine myoblasts).

### In vitro conditioned media (CM) metabolic analysis

C2C12 cells were plated into Agilent Seahorse XF24 (Agilent Technologies, California, USA) plates and differentiated by confluence for 3 days. Meanwhile, EpH4-EV, EO771, NF639, and C2C12 cells were plated at approximately 15% confluence in separate 10 cm dishes for 72 h. The 72-h CM was then removed from all cell lines, centrifuged at 1500 RPM for 10 min, and then the supernatants were collected, diluted 1:3 in fresh growth media, and applied to the differentiated C2C12 cells in Seahorse assay plates for 48 h (*n* = 10 wells per treatment condition) prior to conducting the Agilent Seahorse XF Cell Mito Stress Test protocol according to manufacturer’s instructions.

### In vitro CM PPAR-reporter assays

HEK293 cells were transfected with PPRE-H2b-eGFP^48^ (Addgene #84393) using Invitrogen Lipofectamine 3000 (Thermo Fisher), selected with 500 ng µL^−1^ Gibco geneticin (Thermo Fisher) for 20 days, and flow-sorted to select the cells expressing GFP. The resulting HEK293-PPRE-H2b-eGFP cell line was plated at approximately 15% confluence in a 24-well plate. The following day, cells were imaged using the BioTek Cytation 5 Cell Imaging Multi-Mode Reader (Agilent Technologies) to collect baseline GFP intensity, and 72-hour CM from HEK293, EpH4-EV, EO771, and NF639 cell lines was applied to the 24-well plate, using individual wells as biological replicates (*n* = 6 wells per treatment condition). Cells were then incubated in the CM under normal culture conditions for 24 h, at which point GFP intensity was measured using identical imaging settings as the baseline collection. Mean cellular GFP intensity per well was calculated using Gen5 Microplate Reader and Imager Software (Agilent Technologies) after background flattening and thresholding, which were set consistently across all images.

### Statistical analyses

Patients’ trends of weight change over time were calculated for each individual patient by fitting a simple linear regression line to their weight at each date in the EMR, normalized such that each patient’s first weight record equaled 100, resulting in a slope representing their approximate percentage of body weight change per day. Nineteen patients were identified as outliers in terms of daily weight change. Thirteen of these patients were excluded due to having a limited observation period (<15 days). Each patient’s first albumin measurement was then obtained and the rate of daily weight change was regressed on the patients’ first albumin measurements. Pearson’s correlation coefficients and *p* values were calculated and plotted using *ggpubr*^49^ in R v3.6.1^50^.

Logistic regression analysis was utilized to determine whether serum albumin was predictive for a rate of weight change consistent with cachexia (i.e., <−0.027% per day to reach 5% weight loss in 6 months^1,2^). Omnibus model fit was assessed by chi-square test and effect size was calculated using Nagelkerke’s pseudo-*R*^2^. The receiver operating characteristic curve (ROC) test was conducted, with preferred sensitivity and specificity >0.7. No point on the ROC satisfied these conditions. The fitted logistic regression model was used to predict whether each patient would exhibit a rate of weight loss consistent with cachexia, and these predictions were compared to the actual data to create a confusion matrix for determination of sensitivity, specificity, and positive predictive value.

A multivariate regression model was used to assess clinical data against the 3D-MDS representing overall skeletal muscle gene expression, obtained as described above within “RNA-Sequencing.” Each clinical variable available was regressed on the 3D-MDS and model fit was assessed via MANOVA and Pillai’s trace. Forward selection was then applied to combine the variables with smallest *p* values for Pillai’s trace into a final model, with final selection considerations including statistical significance for MANCOVA using Pillai’s trace and Wilks’ lambda, maximizing Pillai’s trace test statistic, and minimizing Wilks’ lambda test statistic.

Differential gene expression analysis was conducted using *DESeq2*^35^. Input data consisted of transcript-level abundance estimates from Salmon summarized to the gene level using *tximport*^31^. Two differential expression analyses were run: one comparing the group of BC patients to control patients, and another comparing the BC patients by subtype to control patients, with the null hypothesis rejected at FDR < 0.10. Because molecular subtype was the only variable that yielded statistical significance for predicting gene expression in the multivariate regression model described above, no clinical characteristics were assessed in the differential expression analysis.

Differential protein expression analysis was conducted using *DEP*^51^. Protein expression values were first filtered to remove known contaminants and proteins with missing expression values in more than one sample per group, then normalized, and then background-corrected using variance stabilizing transformation. Remaining missing values were imputed using k-nearest neighbor, after determining that the small number of missing values were likely missing at random. Differential expression analysis using linear models and empirical Bayes statistics was then conducted on the imputed dataset, with the null hypothesis rejected at FDR < 0.05.

Input data for GSEA included normalized gene expression counts for each patient for all genes with at least one CPM in at least three patients, with ranking conducted within GSEA using “Signal-to-noise” based ranking. Reference gene sets included all KEGG v7 pathways accessed from Molecular Signature Database^52^ with at least 15 genes in the pathway in our gene set for RNA-seq results and at least 10 genes in the pathway in our gene set for proteomics. Only pathways identified as enriched at *q*-value < 0.05 are reported here. Input data for Enrichr^53,54^ analyses included only the list of significantly DEGs and proteins. In Enrichr, all databases were queried, with particular interest in ENCODE and ChEA Consensus TFs for transcription factor target enrichment, WikiPathways 2019 Human for pathway analysis, and 2018 databases from the Gene Ontology project for cellular component, biological process, and molecular function enrichment analyses. Data presented in this article reflect analyses conducted in Enrichr between May 15 and June 15 of 2019 and in GSEA between March 1 and March 30, 2020. Ingenuity Pathway Analysis (Qiagen, Germany) was utilized for visualization of affected pathways.

ATP content in each mitochondrial subpopulation in the PDOX muscle was compared to control using the Wilcoxon rank sum test. The null hypothesis was rejected at *p* < 0.05. In the CM metabolic experiments, one-way ANOVA was used to compare the rate of oxygen consumed in ATP production as a percentage of basal oxygen consumption between the four CM treatment groups followed by two-tailed Student’s *t* tests with Holm–Bonferroni correction comparing each treatment group to CON Muscle. The null hypothesis was rejected at *p* < 0.05. This experiment was conducted as reported twice with similar results, and results from the first analysis are reported. In the PPAR responsive reporter assays, mean GFP intensities in each well were normalized to account for differences in baseline GFP expression between wells. Normalized GFP intensities at 24 h were compared to normalized baseline measurements using a paired samples two-tailed *t* test by treatment group with Holm–Bonferroni correction for multiple comparisons. The null hypothesis was rejected at *p* < 0.05. This experiment was conducted as reported twice with similar results, with results from the first analysis reported. This analysis includes *n* = 5 for NF639-treated cells due to a technical problem during baseline image capture that resulted in the loss of one image.

For chi-square analyses, the actual number of unique and shared DEGs and dysregulated pathways were initially counted for each subtype. For example

HER2-unique = genes differentially expressed only in HER2 patients

$${\mathrm{HER2}} - 2 - {\mathrm{Group}} = {\mathrm{HER2}}\, \cap \,{\mathrm{ERPR}}\quad \quad + \,{\mathrm{HER2}}\, \cap \,{\mathrm{TP}}\quad \quad + \,{\mathrm{HER2}}\, \cap \,{\mathrm{TN}};$$

$${\mathrm{HER2}} - 3 - {\mathrm{Group}} = {\mathrm{HER2}}\, \cap \,{\mathrm{ERPR}}\, \cap \,{\mathrm{TP}}\, + \,{\mathrm{HER2}}\, \cap \,{\mathrm{ERPR}}\, \cap \,{\mathrm{TN}}\, + \,{\mathrm{HER2}}\, \cap \,{\mathrm{TN}}\, \cap \,{\mathrm{TP}}.$$

Then, expected numbers of DEGs and dysregulated pathways were calculated under the null hypothesis that DEGs and dysregulated pathways are independent of BC subtype. Chi-square statistics were calculated using the difference between observed and expected numbers of DEGs and dysregulated pathways. On 6 degrees of freedom, the chi-square critical value is 12.592 for statistical significance at *α* = 0.05. Bar plots represent the log-fold change in number of DEGs or dysregulated pathways compared to expected values, by category, and actual chi-square statistics for each category are also provided.

For box-and-whisker plots, the width of the box represents the interquartile range (IQR) and whiskers extend to the single extreme measurement in both directions, unless the extreme measurement is considered an outlier, in which case the extreme value is represented as a dot without a connected whisker. Median values are represented by the horizontal line through each boxplot. Outliers in this context are defined as values more extreme than 1.5 × IQR.

### Reporting summary

Further information on research design is available in the Nature Research Reporting Summary linked to this article.

## Supplementary information

Supplementary Information

Supplementary Data 1

Supplementary Data 2

Supplementary Data 3

Reporting Summary

## Data Availability

The processed RNA-Seq and proteomics datasets generated during this study, are publicly available in the figshare repository as part of this figshare data record: 10.6084/m9.figshare.12248951^16^. The dataset ClinicalCharacteristics.xlsx is not publicly available in order to protect patient privacy, but will be made available on reasonable request from the corresponding author. At the time that we formulated our IRB Consent Form and started collecting muscle biopsies (December 2015), we did not have the foresight that the raw transcriptomic and proteomic data would need to be made available publicly. Our IRB Consent Form does not contain language pertaining to this, and therefore, the patients did not give consent to having their genetic information made publicly available. Reconsenting the patients in our study is logistically difficult, if not impossible, since some of our original patients may have completed treatment and are not visiting the Cancer Institute currently, or unfortunately may not have survived to this date. For these reasons, the raw transcriptomic and proteomics data are not publicly available. We acknowledge that this was an unfortunate error on our part. Raw RNA-Seq and proteomics data will be made available on reasonable request from the corresponding author, to researchers who have completed a Data Usage Agreement. Corresponding author details are: Dr. Emidio E. Pistilli, West Virginia University School of Medicine, email address: epistilli2@hsc.wvu.edu.
