# Supplementary material for: Skeletal muscle reprogramming by breast cancer regardless of treatment history or tumor molecular subtype
Source: NPJ Breast Cancer. 2020 Jun 4;6:18. doi: 10.1038/s41523-020-0162-2 (PMC7272425; doi:10.1038/s41523-020-0162-2)
Supplement: Supplementary file 1 — Supplementary Information [file 41523_2020_162_MOESM1_ESM.pdf]

Supplementary Figures

Supplementary Figure 1. Scaled, log-transformed protein expression correlated with scaled, log-transformed RNA expression in a gene-wise fashion, presented for 8/8 patients with both RNA seq and proteomic analyses.

Supplementary Figure 2. GSEA using proteomic data from muscle biopsies from n=5 women with HER2-overexpressing tumors and n=5 control patients.

Supplementary Figure 3. Full uncropped western-blots used to make Figure 5.

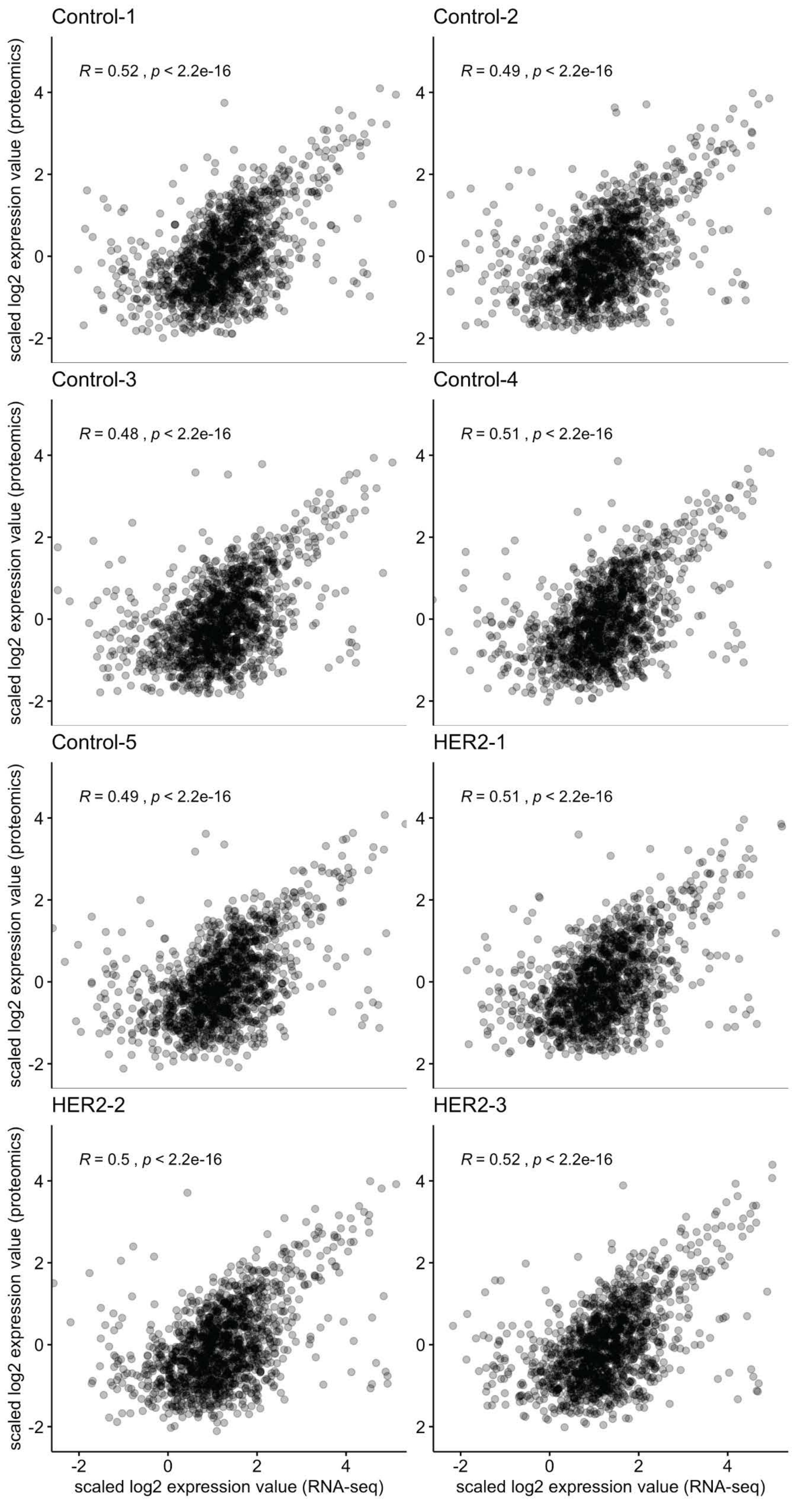

Control-1
R = 0.52 , p < 2.2e-16
Control-2
R = 0.49 , p < 2.2e-16
Control-3
R = 0.48 , p < 2.2e-16
Control-4
R = 0.51 , p < 2.2e-16
Control-5
R = 0.49 , p < 2.2e-16
HER2-1
R = 0.51 , p < 2.2e-16
HER2-2
R = 0.5 , p < 2.2e-16
HER2-3
R = 0.52 , p < 2.2e-16
scaled log2 expression value (proteomics)
scaled log2 expression value (RNA-seq)

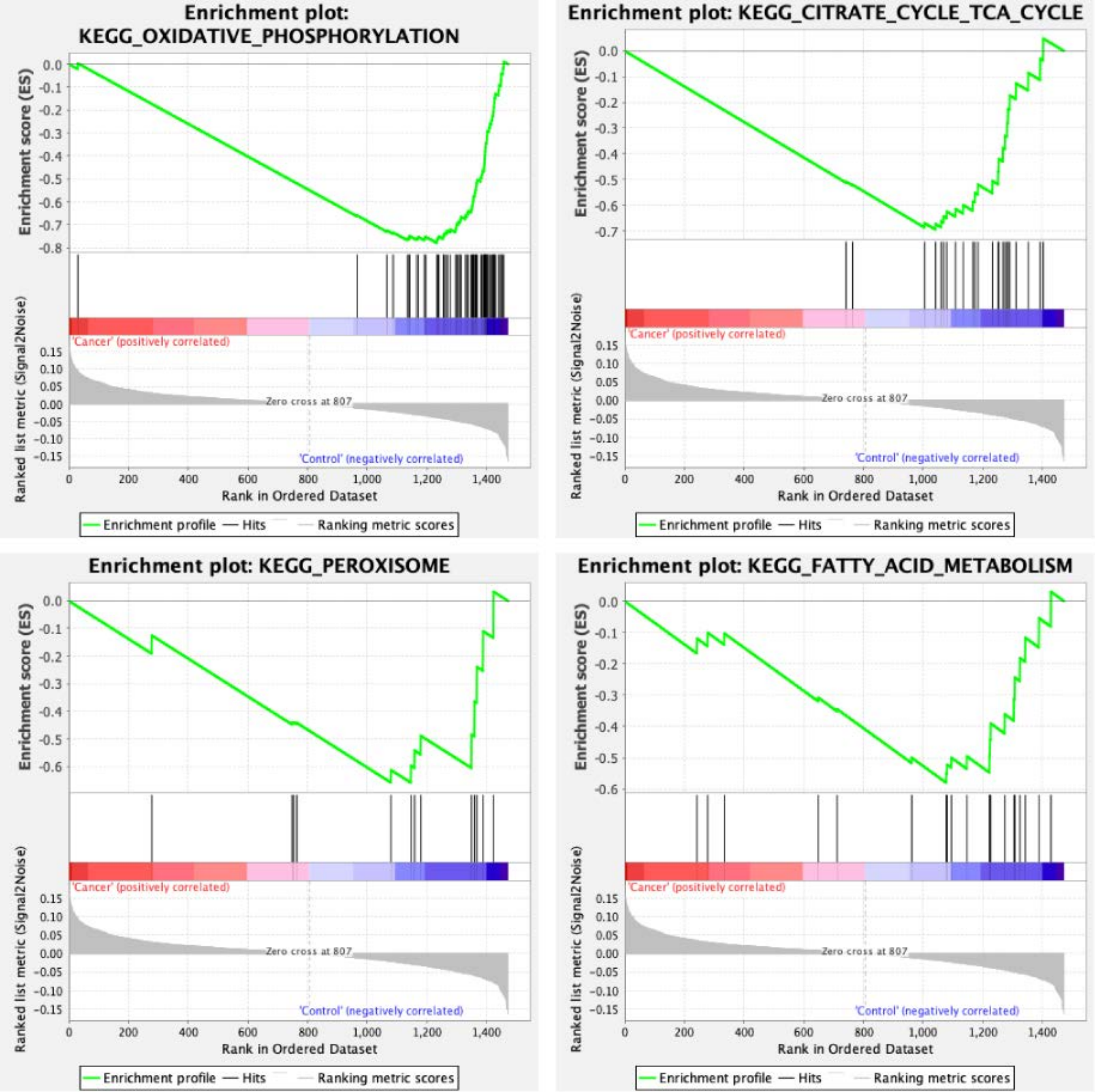

Enrichment plot: KEGG_OXIDATIVE_PHOSPHORYLATION
Enrichment score (ES)
'Cancer' (positively correlated)
Zero cross at 807
'Control' (negatively correlated)
Ranked list metric (Signal2Noise)
Rank in Ordered Dataset
Enrichment profile
Hits
Ranking metric scores
Enrichment plot: KEGG_CITRATE_CYCLE_TCA_CYCLE
Enrichment score (ES)
'Cancer' (positively correlated)
Zero cross at 807
'Control' (negatively correlated)
Ranked list metric (Signal2Noise)
Rank in Ordered Dataset
Enrichment profile
Hits
Ranking metric scores
Enrichment plot: KEGG_PEROXISOME
Enrichment score (ES)
'Cancer' (positively correlated)
Zero cross at 807
'Control' (negatively correlated)
Ranked list metric (Signal2Noise)
Rank in Ordered Dataset
Enrichment profile
Hits
Ranking metric scores
Enrichment plot: KEGG_FATTY_ACID_METABOLISM
Enrichment score (ES)
'Cancer' (positively correlated)
Zero cross at 807
'Control' (negatively correlated)
Ranked list metric (Signal2Noise)
Rank in Ordered Dataset
Enrichment profile
Hits
Ranking metric scores

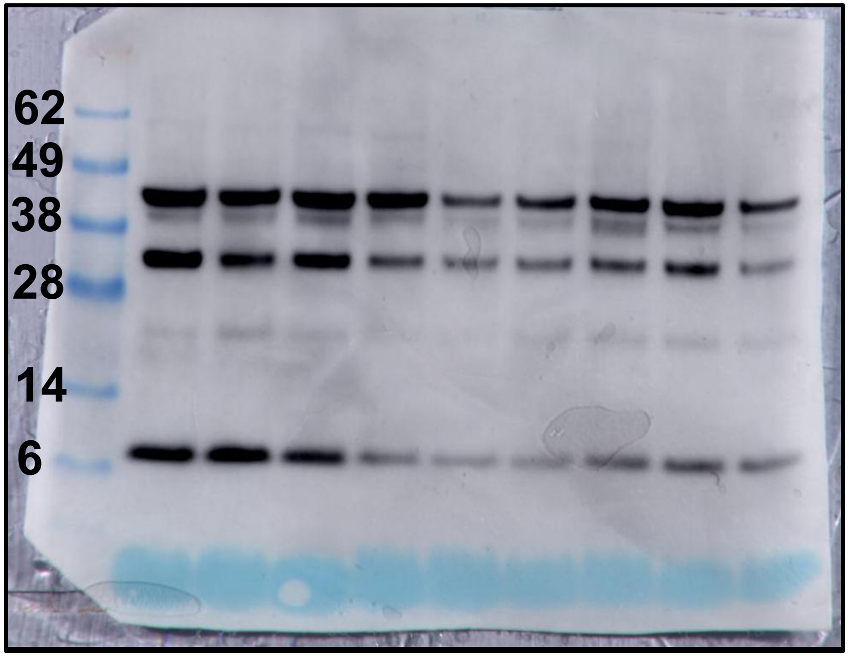
62
49
38
28
14
6
CKMT2 (48kDa)
COX7A1 (9kDa)

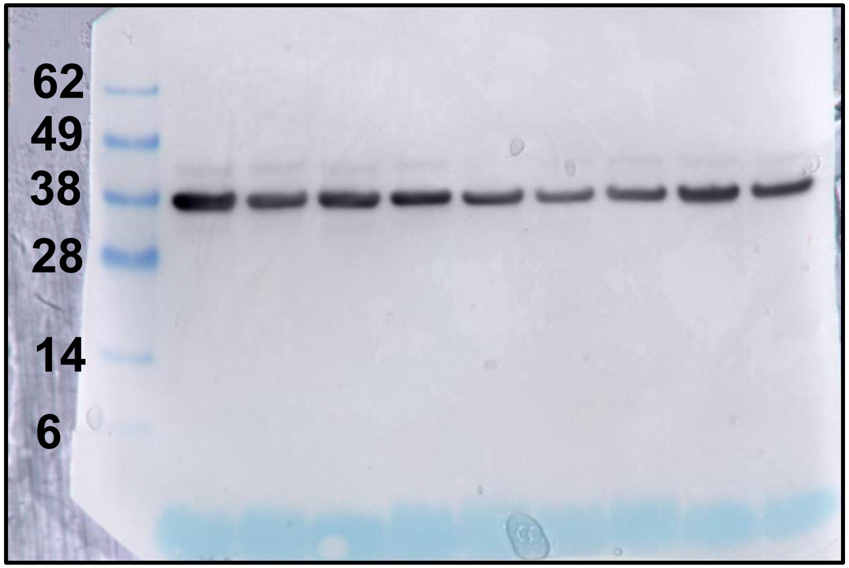
62
49
38
28
14
6
GAPDH (38kDa)
